# Exploration of the clinical prognostic model of BRCA based on PCAT7

**DOI:** 10.3389/fonc.2025.1580858

**Published:** 2025-07-30

**Authors:** Zhi Zhang, Chaocheng Xiang, Tong Chen, Aimin Ma, Xu Wang, Jiaying Li, Yixuan Chen, Chengyu Huang, Ting Li, Danmei Wu, Steven Mo, Dequan Li

**Affiliations:** ^1^ Department of Hepatobiliary Surgery, Guangxi Medical University Affiliated Wuming Hospital, Nanning, China; ^2^ Department of Breast Surgery, Guangxi Medical University Affiliated Wuming Hospital, Nanning, China; ^3^ Experimental Center of BIOQGene, YuanDong International Academy of Life Sciences, Hong Kong, Hong Kong SAR, China; ^4^ Systems Biology Research Center, Biology Institute, Guangxi Academy of Sciences, Nanning, China

**Keywords:** PCAT7, BRCA, WGCNA, immune cells, PCAT7 clinical model

## Abstract

**Purpose:**

Breast cancer (BRCA) is the most common cancer in women. Overexpression of long non-coding RNA Prostate cancer-associated transcript 7 (PCAT7) in BRCA was correlated with an unfavorable prognosis. Consequently, investigating the function and prognostic significance of PCAT7 in BRCA has become imperative.

**Methods:**

This study used BRCA data from the Cancer Genome Atlas (TCGA) as a training cohort to evaluate the prognostic potential of PCAT7. In addition, luminal A, luminal B, HER2, and basal like triple-negative breast cancer samples were collected clinically to verify the expression of PCAT7. Meanwhile, differentially expressed genes (DEGs) related to PCAT7 were identified. Subsequently, weighted gene co-expression network analysis (WGCNA) was used to identify abnormal regulatory modules of PCAT7 co-expressed genes in BRCA. Furthermore, we used enrichment analysis to evaluate the distribution patterns of genes. We constructed a clinical indicator scoring model based on PCAT7 based prognosis-related genes, followed by correlation analyses to study the relationship between clinical indicators based on PCAT7 expression and immune cell infiltration, immune checkpoint-related genes, and tertiary lymphoid structure marker genes. Pivot analysis based on a hypergeometric approach was used to identify lncRNAs, TFs and RBPs that regulate the set of prognosis-related genes to explore drug targets.

**Results:**

The results showed that PCAT7 was significantly high expression in BRCA, and patients with high expression of PCAT7 had poor prognosis. IHC further confirmed that PCAT7 was significantly overexpressed in BRCA samples of different subtypes, suggesting that PCAT7 has diagnostic potential in BRCA. Meanwhile, a total of 28,892 DEGs and 954 DEmiRNAs were continuously upregulated or downregulated. The most relevant module genes associated with PCAT7 are significantly enriched in immune and cancer-related pathways. PCAT7-based models and model genes were significantly associated with multiple immune checkpoint-related genes and tertiary lymphoid structure marker genes. In addition, PCAT7 is associated with the inhibition of immune cell infiltration.

**Conclusion:**

We found that the clinical score of PCAT7 is significantly correlated with the prognosis of BRCA patients, suggesting that PCAT7 is a useful biomarker.

## Introduction

Breast cancer (BRCA) is the most common malignant tumor in the world and the main cause of cancer death. There are many causes of BRCA, such as excessive nutritional intake, a high-fat diet, obesity, and excessive drinking, which increase the incidence of cancer (1). In addition, women have many other risk factors for developing BRCA, but the most notable risk factors are age, genetic factors and estrogen exposure (2). In the past, we have studied the treatment of BRCA, but there are still many barriers to treating this disease due to the heterogeneity of the disease at the molecular and genetic levels (multiple subtypes). In addition, due to the heterogeneity of BRCA, several traditional classification criteria, such as tumor size, axillary lymph node size, histological grade, and steroid receptor expression level, have been established. Histologic stratification of BRCA, for example, is based primarily on the expression of progesterone receptor (PR), estrogen receptor (ER), and human epidermal growth factor receptor 2 (HER2). This is the basis for the classification of BRCA (3), which has luminal A, luminal B, HER2-enriched, basal-like and normal-like subtypes (4, 5). In addition to the molecular heterogeneity, the treatment of breast cancer still faces many practical difficulties, such as: The strength gap between countries was significant, and BRCA patients in underdeveloped countries do not have good resources (6); Early onset was difficult to diagnose, resulting in missing the optimal surgical resection stage in the later stage (7); The elderly lack the awareness of early medical treatment, resulting in a high incidence rate of BRCA (8) in elderly individuals. Therefore, there are still many difficulties to overcome in BRCA treatment.

With the continuous progress of related research, people are gradually shifting from treatment decisions mainly based on the degree of disease anatomy to potential biological mechanisms. The utilization of gene array technology has highlighted the heterogeneity of BRCA, which consists of various biological subtypes, while enabling the prediction of chemotherapy response through gene analysis. Therefore, further research on BRCA could be beneficial for revealing new and promising prognostic biomarkers and drug targets, thereby improving the clinical efficacy of cancer treatment. Long noncoding RNA (lncRNA) is a protein-free RNA molecule with a length of more than 200 nucleotides that regulates the occurrence and development of various malignant tumors, including cancer (9). The lncRNA Prostate cancer-associated transcript 7 (PCAT7), specifically the lncRNA Prostate Cancer Related Transcript 7, spans 1,937 base pairs and is located at chromosomal region 9q22.32. It is recognized as a cancer-associated gene. Studies have demonstrated that PCAT7 is highly expressed in numerous malignant tumors, including lung cancer, BRCA, and prostate cancer, and has been associated with unfavorable prognoses (10–12). Abnormal expression of PCAT7 in BRCA tissues and cells promotes cancer cell proliferation, migration, and invasion while inhibiting apoptosis, and promotes malignant progression of breast cancer by regulating the expression of a range of downstream genes (12). At present, further exploration of BRCA dysregulation genes related to PCAT7 overexpression has not been conducted, and these genes may also play important roles in diseases, which is worthy of further exploration.

This study aimed to explore the mechanism of action of the PCAT7 gene in the occurrence and development of BRCA, providing a basis for accurate diagnosis and targeted treatment of this disease. We performed weighted gene coexpression network analysis (WGCNA) on the dysregulated genes influenced by high expression of PCAT7 in BRCA. The PCAT7 clinical scoring model was further developed based on the module genes with the highest correlation, revealing the multiomics patterns of the global regulatory network.

## Materials and methods

### Source of organization

Total 32 breast samples were collected from Wuming Hospital Affiliated to Guangxi Medical University, including 12 control cases, 5 cases of luminal A, 5 cases of luminal B, 5 cases of HER-2(3+) and 5 cases of triple-negative breast cancer (TNBC). This study has been approved by the Ethics Review Committee of Wuming Hospital affiliated to Guangxi Medical University. Studies involving human participants were in accordance with the Declaration of Helsinki, and were reviewed and approved by the Ethical Review Board of Wuming Hospital affiliated to Guangxi Medical University (WM-2024(163)), with samples obtained from all patients with informed consent.

### Data resource collection

The relevant data and clinical information for patients with BRCA were obtained from The Cancer Genome Atlas (TCGA, https://www.cancer.gov/) database. The expression profile included 1,218 samples, including 1,104 BRCA tissue samples and 113 adjacent cancer samples (as the control group of this study). The data were subsequently normalized using the limma package (13), and the distributions of BRCA tissue samples and control samples were visualized through principal component analysis (PCA).

### IHC staining evaluation

Sections were deparaffinized and hydrated, followed by exposure of antigenic sites by microwave antigen repair. Subsequently, after washing using PBS, sections were placed in 3% H_2_O_2_ to block endogenous peroxidase activity. After blocking with serum, sections were incubated with primary antibodies overnight at 4°C and with secondary antibodies for 30 min at room temperature the following day. After DAB coloration, the sections were observed to be brown in color for the target protein under the microscope, while the nuclei were blue in color by hematoxylin staining. Finally, the sections were dehydrated, transparent, and sealed for further observation under the light microscope. The positive results show variable shades of brown, whereas the nuclei are blue.

### Diagnosis and prognostic effect of the PCAT7 gene in BRCA

We used the R package pROC (14) to evaluate the potential of PCAT7 as a diagnostic marker for BRCA. Subsequently, we classified BRCA patients into high-expression and low-expression groups of PCAT7 based on the median expression level of PCAT7. We utilized the Kaplan–Meier method (15) to perform survival analysis, which illustrated the association between PCAT7 expression and patient survival in patients with BRCA. Survival analysis was performed through the logarithmic rank test, and an adjusted p value (*p*. adjust< 0.05) indicated a statistically significant difference.

### Differential gene expression analysis

To investigate the dysregulated genes associated with high expression of PCAT7 in BRCA, we utilized the limma package (13) to analyze differential gene expression and identify potential disease-related genes. Differentially expressed genes (DEGs) and miRNAs (DEmiRNAs) with *p*.adjust <0.05 were considered significant. In both the RNA and miRNA sets, we identified genes that were consistently upregulated or downregulated; these genes were considered dysregulated genes associated with high expression of PCAT7 in BRCA.

### Weighted gene coexpression network analysis

The DEmRNAs associated with high PCAT7 expression in BRCA were subjected to WGCNA using the “WGCNA” R package to identify relevant co-expression modules. Then, we constructed a scale-free network and applied the gradient method to assess the scale independence and average connectivity of these modules. By setting the independence threshold to 0.85, we selected the most suitable power value to establish a scale-free gene coexpression network. We considered high PCAT7 expression a clinical factor and used heatmaps to visualize the relationships between different modules and clinical factors. A high correlation indicates that the genes in the corresponding modules frequently exhibit a strong association with disease status. In this study, the module most significantly positively correlated with BRCA was selected as the research module.

### Functional enrichment analysis

To gain a comprehensive understanding of the biological functions associated with PCAT7, we performed Gene Ontology (GO) and Kyoto Encyclopedia of Genes and Genomes (KEGG) enrichment analyses of the BRCA module genes related to PCAT7 using the clusterProfiler package (16). *p* values < 0.05 were used to indicate significant enrichment of functions or pathways.

### Gene set enrichment analysis

We used genomic enrichment analysis (GSEA) (17) to identify meaningful biological features of the high- and low-expression PCAT7 groups in BRCA. The analysis was based on the Molecular Feature Database (MsigDB) (https://ngdc.cncb.ac.cn/databasecommons/database/id/1077) of c5.bp.v7.0/entrez.gmt and c2.cp.kegg.v7.0.symbols.gmt as internal parameter genomes. The analysis was performed using the clusterProfiler package developed in the R language. The enrichment results were considered significant when the p value was < 0.05.

### Construction and validation of a clinical model using PCAT7 scores

Initially, this study performed recurrence-free survival (RFS) and overall survival (OS) analyses of BRCA patients by employing the module genes that are most strongly associated with PCAT7. The top 50 genes significantly associated with BRCA prognosis and PCAT7 expression were selected as scoring genes, and further Cox univariate and multivariate analyses were performed to construct a PCAT7-based scoring model based on these genes to assess their prognostic effects in BRCA patients.

Univariate Cox regression analysis was first performed to identify mRNAs significantly associated with prognosis in BRCA patients. mRNAs significantly correlated with independent prognostic factors were considered meaningful and included in a multivariate Cox regression to construct a prognostic model. The prognostic index (PI) was calculated using the formula:


$$\text{PI}={\text{Expr}}_{\text{mR}1}{\text{ β}}_{\text{mR}1}+{\text{ Expr}}_{\text{mR}2}{\text{ β}}_{\text{mR}2}+{\text{ Expr}}_{\text{mR}3}{\text{ β}}_{\text{mR}3}\text{……}$$

Where β represents the regression coefficient and Expr denotes the expression level of each mRNA. This model was used to evaluate prognostic risk and determine whether it serves as an independent prognostic factor. Finally, candidate mRNAs were analyzed together with clinical features using univariate and multivariate Cox regression to assess their predictive value for OS and RFS.

The ability of the model to predict the prognosis of BRCA patients was subsequently evaluated by generating nomogram plots and calibration curves, which were generated based on the rms package (18).

### Immunocyte infiltration analysis

In this study, we analyzed the abundance of immune cells in BRCA samples using CIBERSORT (https://cibersort.stanford.edu/). The extent of immune cell infiltration was assessed in the PCAT7 control, high expression, and low-expression groups. In addition, correlation analysis was used to explore the correlation between PCAT7 expression, PCAT7-based scoring model genes, immune cell infiltration, immune checkpoint-related genes, and markers of tertiary lymphatic structure.

### Identification of upstream regulators

The RNAInter, TRRUST, STRING and DrugBank databases were used as background sequences for this study. The expression of regulatory genes was regulated by lncRNAs, transcription factors (TFs) and RNA-binding proteins (RBPs) through a hypermetric study of gene sets based on the PCAT7 scoring model. The results of the analysis of differential gene expression were used to identify lncRNAs that were differentially expressed, and then, Pivot analysis based on a hypergeometric approach was used to identify lncRNAs, TFs, and RBPs that regulate the scoring gene set to explore drug targets.

### A multidimensional landscape gene regulation scoring model based on PCAT7

To elucidate the single nucleotide polymorphisms (SNPs) of genes in BRCA based on the PCAT7 scoring model, the mutation status and details of the genes were visualized using the R package maftools (19), and the copy number variations (CNVs) of these genes in BRCA patients were determined. In addition, we explored the correlation between gene methylation levels and transcriptional levels via clinical scoring via correlation analysis methods based on the PCAT7 clinical scoring model.

### Data analysis and statistics

All bioinformatics analyses in this study were conducted via the BioinforCloud platform (http://www.bioinforcloud.org.cn).

## Results

### High expression of PCAT7 in BRCA mediates poor prognosis

**Figure 1:**
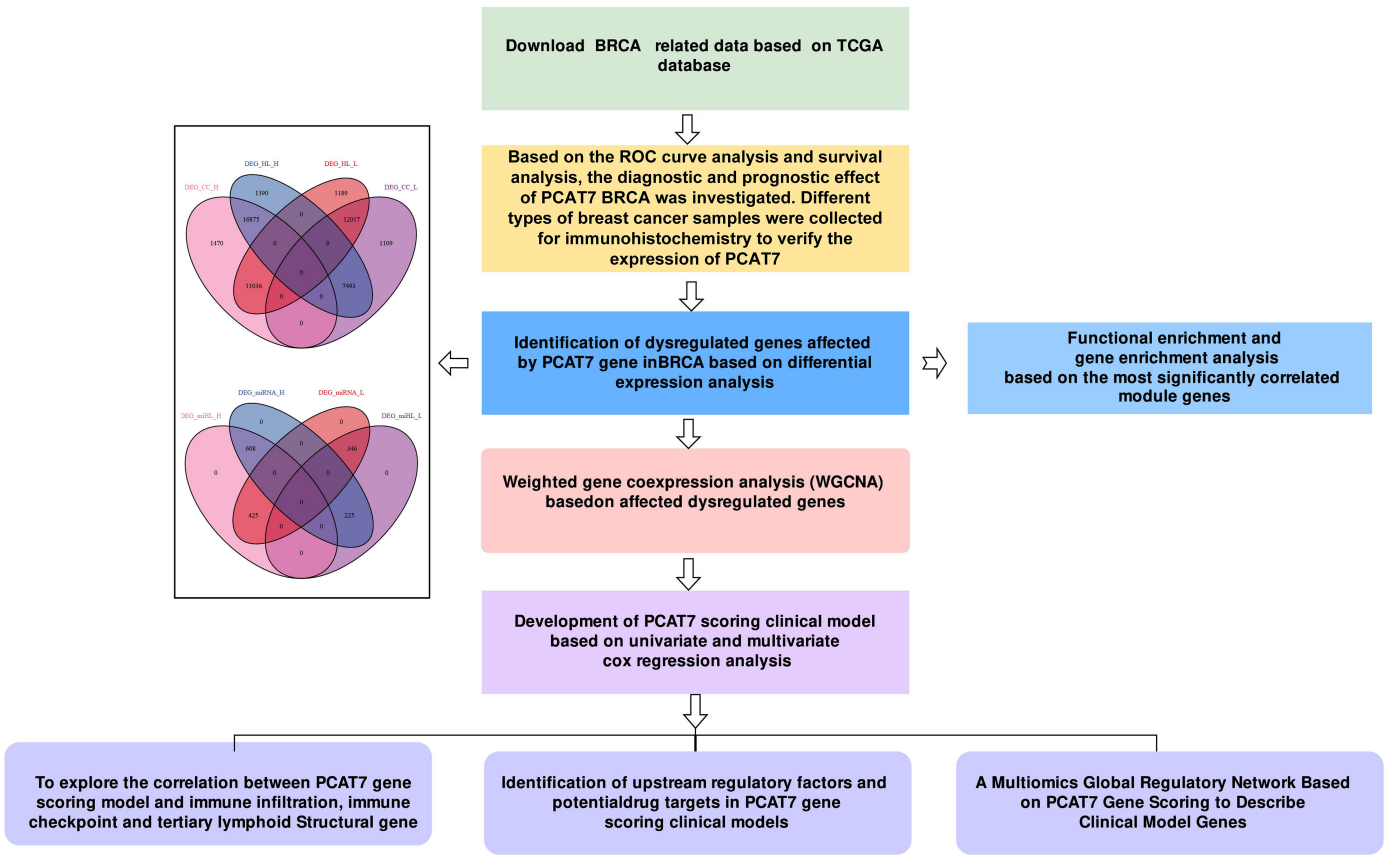
Workflow diagram. This study was divided into five parts. First, we used the TCGA database to download data related to breast cancer (BRCA) and carried out subject operating characteristic (ROC) curve analysis and survival rate analysis. In the second part, we identified dysregulated genes associated with PCAT7 through differential expression analysis and conducted weighted gene coexpression network analysis (WGCNA) and enrichment analysis. Next, we constructed a PCAT7-based scoring clinical model based on Cox regression analysis, explored the relationships of the scores and genes related to prognosis and clinical indicators, and conducted a gene ontology (GO) semantic similarity analysis. Finally, we investigated the relationships between the PCAT7-based clinical model and the immune microenvironment, upstream regulatory factors, and potential drug targets and constructed a multiomics global regulatory network.

**Figure 2:**
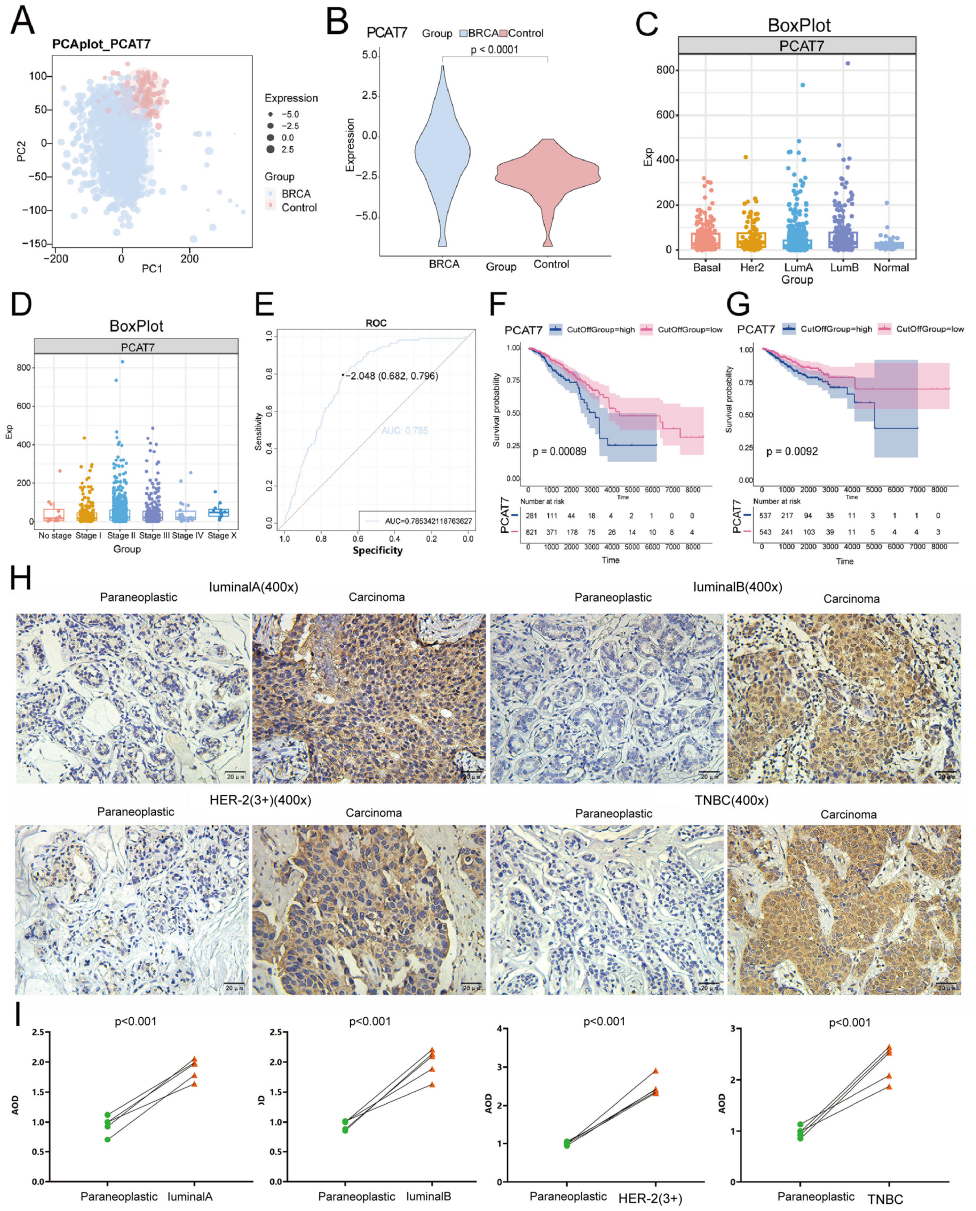
PCAT7 gene expression imbalance in breast cancer. **(A)** Principal component analysis density map mapping of PCAT7 expression. **(B)** Violin Chart Showing PCAT7 Transcription Levels in Cancer Patients. **(C)** Box plots show PCAT7 expression in controls and different BRCA types. **(D)** Box plots show PCAT7 expression at different stages of BRCA. **(E)** Subject operating characteristic (ROC) curve showing the potential of PCAT7 for cancer diagnosis. F-G. Survival curves indicating the prognostic potential of overall survival (OS) **(F)** and recurrence-free survival (RFS) **(G)** in BRCA patients. **(H)** IHC staining verified the expression levels of PCAT7 in lumen A, lumen B, HER2, and basal-like trinegative breast cancer (TNB). Scale bar is 400 μm. **(I)** Intensity of protein expression of PCAT7 in PCAT7 in lumen A, lumen B, HER2 and TNB tissues and non-cancerous breast tissues.

The flow of this study is shown in **Figure 1**. The PCA method was used to reduce the dimension of the data to obtain a two-dimensional scatter plot (**Figure 2A**). PCAT7 was highly expressed in the BRCA group, as compared with PCAT7 transcript levels in controls (**Figure 2B**). PCAT7 expression was higher in luminal A, luminal B, HER2, and basal-like triple negative breast (TNB) cancers compared with controls (**Figure 2C**). Expression of PCAT7 was increased in different BRCA stages (**Figure 2D**). The ROC curve also showed that PCAT7 had a certain diagnostic efficacy for BRCA and was a potential BRCA diagnostic marker (AUC=0.785) (**Figure 2E**). Furthermore, in the analysis of OS and RFS, high expression of PCAT7 was significantly associated with shorter OS and RFS in BRCA patients (**Figures 2F, G**). PCAT7 staining was higher in BRCA luminal A, luminal B, HER2, and TNB breast cancer tissues than in controls; PCAT7 was mainly located in the cytoplasm of the specimens, and staining was predominant in the tumor tissues (**Figure 2H**). Paired dot plot showed that the expression level of PCAT7 protein in cancer tissues was higher than that in adjacent tissues (*p* < 0.001) (**Figure 2I**). Therefore, studies have shown that high expression of PCAT7 is significantly associated with poor prognosis in BRCA patients and has diagnostic potential.

### Expression of related genes characterizing the global regulatory mode of PCAT7 in BRCA

**Figure 3:**
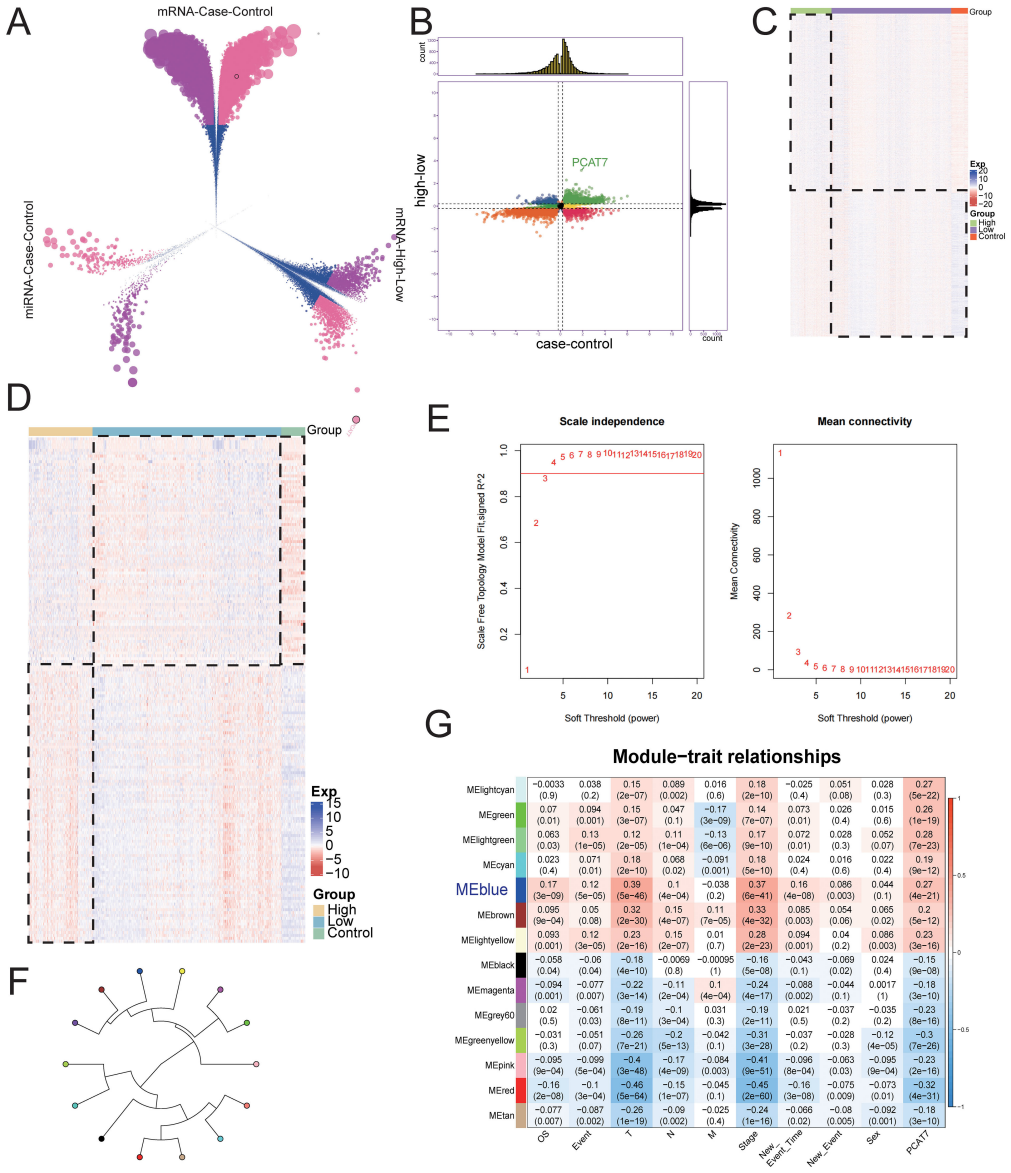
The global regulatory mechanism of the PCAT7 gene in BRCA. **(A)** Volcano plot showing differentially expressed genes (DEGs) and differentially expressed miRNAs (DEmiRNAs) in the control group and breast cancer (BRCA) cohort and in the PCAT7 high-expression group and low-expression group. **(B)** Nine-piece grid scatter plot showing differentially expressed genes and miRNAs in BRCA patients affected by PCAT7. **(C)** Heatmap showing the expression of the maladjustment gene control and high low-grouping genes in breast cancer patients affected by PCAT7. **(D)** Heatmap showing the expression of control and high-low group genes related to PCAT7-related disorders in patients with breast cancer. **(E)** Selection of the soft-thresholding powers. The left panel showing the scale-free fit index versus soft-thresholding power. The right panel displays the mean connectivity versus soft-thresholding power. **(F)** The module loop tree displays the connectivity of PCAT7 coexpression modules. **(G)** The correlations between the gene coexpression module and PCAT7 and clinical features are displayed through a module correlation heatmap.

By analyzing differential gene expression, BRCA and control groups were identified to have differential expression genes (DEGs) and miRNAs (DEmiRNAs). The criteria for identifying significantly differentially expressed genes were adjusted p value < 0.05 and absolute log fold change > 0.2. This includes 18,345 up-regulated and 13,126 down-regulated DEGs, 608 up-regulated DEmiRNAs, and 346 down-regulated DEmiRNAs (**Figure 3A**). From the comparison of high and low expression groups of BRCA, 18,265 up-regulated and 13,206 down-regulated DEGs, 608 up-regulated and 346 down-regulated DEmiRNAs were obtained. Furthermore, in the differential results of the two groups, 28,892 continuously up-regulated or down-regulated DEGs and 954 continuously up-regulated or down-regulated DEmiRNAs were also discovered, defined as dysregulated genes and DEmiRNAs associated with high expression of PCAT7 in BRCA (**Figure 3B**). The expression dysregulated genes and miRNAs related to PCAT7 can be visually displayed in heatmaps across control group, high expression group of PCAT7, and low expression group (**Figures 3C, D**).

Based on the evaluation of the scale-free topology fit index and mean connectivity, the soft-thresholding power was set to 3 (**Figure 3E**). Subsequently, a WGCNA results, genes with similar expression patterns were clustered using dysregulated genes affected by high expression of PCAT7, resulting in a total of 14 expression modules (**Figure 3F**). Further calculations were conducted to determine the correlation between each module and PCAT7 expression, as well as clinical features such as clinical models. A significant positive correlation was found between the blue module and the PCAT7 gene (r=0.39 *p*=5e-46) (**Figure 3G**). Therefore, the blue module may be a key module mediating poor prognosis in PCAT7 patients, and further research is needed.

### Biological functions and signaling pathways significantly regulated by PCAT7-induced dysregulation of BRCA genes

**Figure 4:**
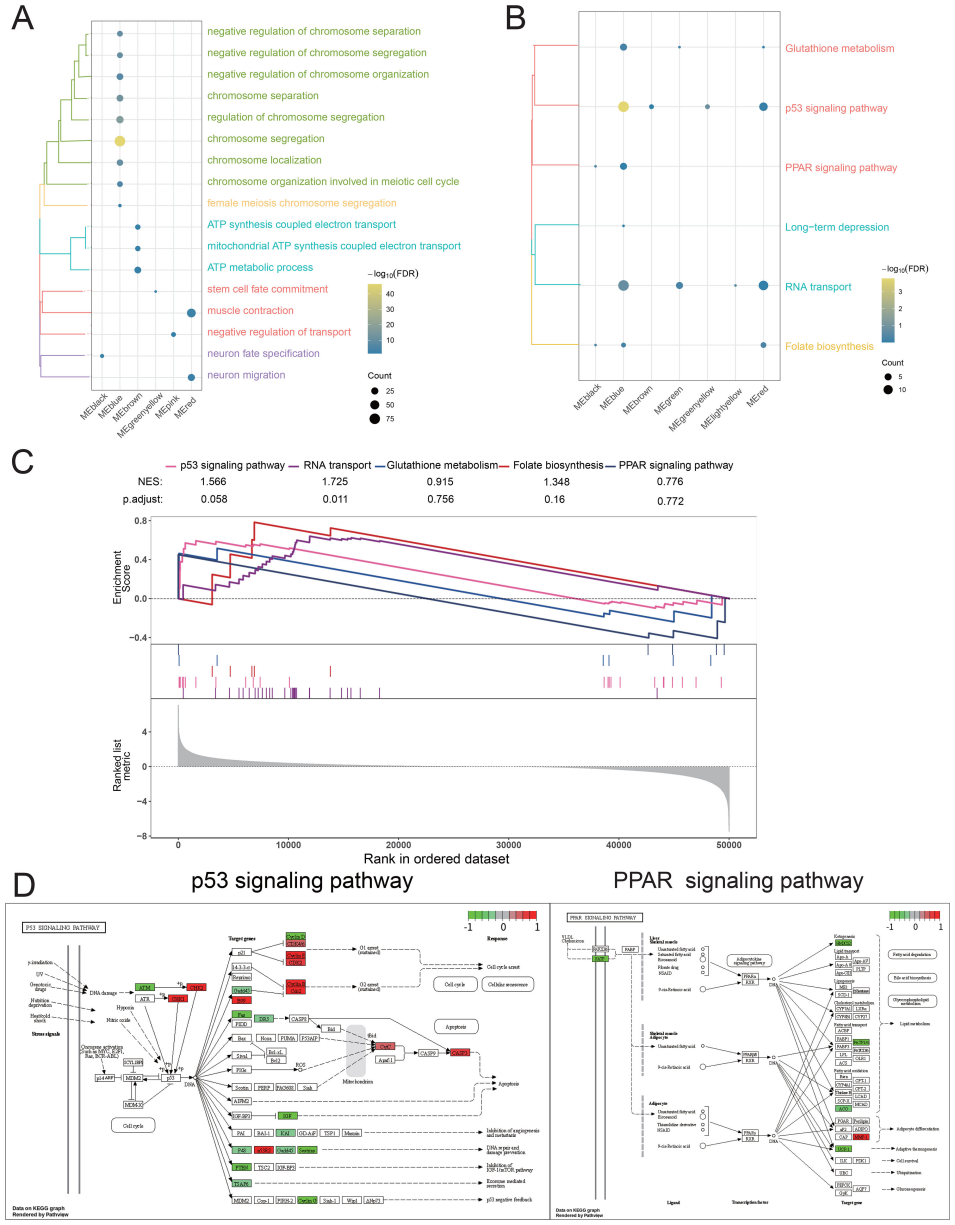
Biological function and signaling pathways significantly regulated by the PCAT7 gene in patients with breast cancer. **(A)** Cluster bubble chart showing the biological processes associated with the significant regulation of the PCAT7 gene. **(B)** Cluster bubble chart showing the KEGG pathways significantly regulated by the PCAT7 gene. Normalized Enrichment Score (NES): NES indicates whether a gene set is significantly concentrated (i.e., enriched) in high-expression or low-expression phenotypes. The larger the value (positive or negative), the more significant the enrichment. **(C)** GSEA diagram showing the signaling pathways that significantly activate/inhibit the PCAT7 gene. **(D)** Pathway map showing significantly activated/inhibited signaling pathways of the PCAT7 gene.

To further reveal the significance of the disease-related genes, enrichment analysis was conducted on the blue module genes, and the results revealed that these genes significantly participate in BP pathways, such as chromosome separation; negative regulation of chromosome separation; ATP synthesis coupled with electron transport (**Figure 4A**); and KEGG pathways, such as the PPAR signaling pathway, p53 signaling pathway (**Figure 4B**). Furthermore, GSEA further confirmed the activation of KEGG signaling pathways in the blue module, suggesting that these pathways may play an important role in the occurrence and development of BRCA (**Figure 4C**). The pathway map diagram further clarified the expression of genes in the P53 signaling pathway and PPAR signaling pathway (**Figure 4D**). In summary, our research results indicate that the high expression of PCAT7 affects abnormally regulated genes in BRCA, significantly impacting its biological functions, including signaling pathways such as P53 and PPAR, to regulate BRCA progression.

### A prognostic clinical model based on PCAT7 has significant prognostic efficacy

**Figure 5:**
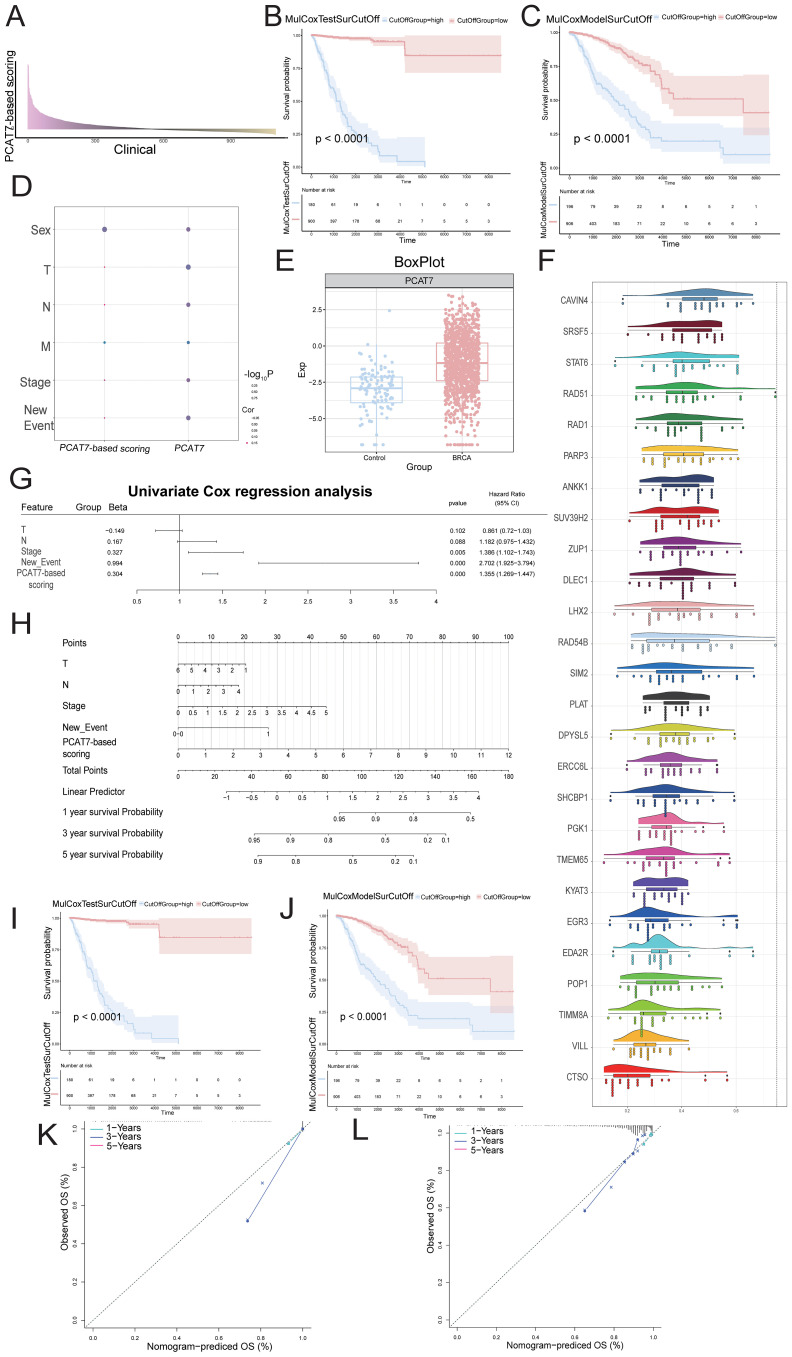
The influence of the clinical model with the PCAT7 score on the prognosis of breast cancer patients. **(A)** The bar graph shows the multivariate Cox score of the gene set related to PCAT7 expression in clinical samples of breast cancer patients. **(B, C)** RFS and OS survival curves based on the PCAT7 score. **(D)** The bubble chart shows the correlation between Cox prognostic score genes and clinical parameters. **(E)** Box diagram showing the transcription level of PCAT7 in breast cancer cells. **(F)** Cloud and rain maps displaying biological associations between the scoring genes. **(G)** Model column chart displaying the clinical model based on the PCAT7 score. **(H)** Forest plot showing the multifactorial prognostic efficacy of the PCAT7 score and clinical indicators. (**I, J)** Survival curve showing the prognostic potential of RFS and OS in clinical models based on the PCAT7 score. **(K, L)** The calibration curve shows the prognostic potential of RFS and OS in clinical models based on PCAT7 scoring.

To investigate the role of the blue module in BRCA, we first conducted RFS and OS survival analyses on patients (**Supplementary Figure S1**), selected the top 50 significant genes and PCAT7 from the analysis results (**Table 1**), and then conducted univariate and multivariate Cox regression analyses. Subsequently, an expression profile was generated for the BRCA clinical cohort for the set of functional genes associated with PCAT7 expression (**Figure 5A**). Survival curve analysis revealed that patients with high PCAT7 index scores had poor prognoses (**Figures 5B, C**). In addition, PCAT7-based scoring genes were associated with primary tumor lesions (T), metastatic lymph nodes (N), and distant metastasis (M) and were significantly correlated with BRCA patients (**Figure 5D**). Subsequently, we calculated and displayed the expression values of the genes in different groups. Subsequently, it was visually observed that the transcript expression levels of the genes in the case group were greater than those in the control group (**Figure 5E**). Additionally, through biological association analysis, we found biological correlations between PCAT7 and PCAT7-based scoring genes (**Figure 5F**). We further constructed a clinical model based on PCAT7 (**Figure 5G**) and analyzed the scores and clinical indicators based on PCAT7 through Cox regression analysis. The results showed that (**Figure 5H**) tumor stage and the PCAT7 score were found to be independent risk factors for BRCA. Survival analyses demonstrated that high scores were significantly associated with poor BRCA prognosis (**Figures 5I, J**). The calibration curve indicated that the model had good predictive accuracy (**Figures 5K, L**). These results suggest that PCAT7 and its related genes play a key role in BRCA, and its high expression predicts poor prognosis.

**Table 1:** Top 50 genes most significantly associated with BRCA prognosis.

Gene	OS_pvalue	RFS_pvalue
PCAT7	0.000894016	0.009220141
AC007686.3	3.84E-06	0.160771094
AC010205.1	4.75E-05	1.82E-06
AC011503.3	0.000323189	0.000663676
AC024884.1	5.11E-05	0.041773229
AC090912.2	9.56E-06	0.003259798
AC091825.3	0.000282405	0.123801645
AC092718.4	0.000356066	0.013932155
AC098934.1	0.000407629	0.017494379
AC098934.2	8.43E-05	0.344365068
AC123912.4	0.000217516	0.055886228
AC136475.1	8.68E-05	0.021149505
AL137779.2	0.000316427	0.02210511
AL139094.1	3.49E-05	0.184007679
AL512378.1	0.000428814	0.001735604
ANKK1	9.29E-05	0.024142284
AP003555.2	0.000241107	0.030548908
BOLA2-SMG1P6	0.000261533	0.231745115
C17orf107	0.00017898	0.029872983
CACYBPP2	0.000155275	0.019682947
CAVIN4	2.72E-05	0.0241482
CTSO	0.000361982	0.001096901
DHRS12	0.000225441	0.135650273
DLEC1	6.08E-05	0.029100176
DPYSL5	0.000340986	0.163684841
EDA2R	9.15E-05	0.114936426
EGR3	0.00031848	0.000380158
ERCC6L	0.000263538	0.058230777
FAM72C	4.87E-05	0.001792222
GARS	8.23E-05	0.004554245
HPDL	3.14E-05	0.000761205
KYAT3	0.000179635	0.002772574
LHX2	0.000428809	0.01428945
PARP3	1.51E-05	0.000358034
PGK1	2.75E-09	0.010121181
PLAT	0.000409736	0.059808118
POP1	1.14E-05	0.01237214
RAD1	0.00038394	0.248829162
RAD51	0.000200105	0.038794608
RAD54B	0.000296975	0.095013678
RNU6-342P	0.000310147	0.004921095
SHCBP1	3.14E-05	0.006356932
SIM2	0.00033271	0.059504852
SRSF5	0.000185114	0.012001966
STAT6	9.99E-05	0.112406798
SUV39H2	0.000275013	0.085503496
TARS	0.00017885	0.031974139
TIMM8A	9.92E-05	0.05549631
TMEM65	1.55E-05	0.00187059
VILL	5.19E-05	0.013667882
ZUP1	0.000137694	0.127013737

### Disrupted expression of the PCAT7-based model gene reprogrammes the immune microenvironment of BRCA

**Figure 6:**
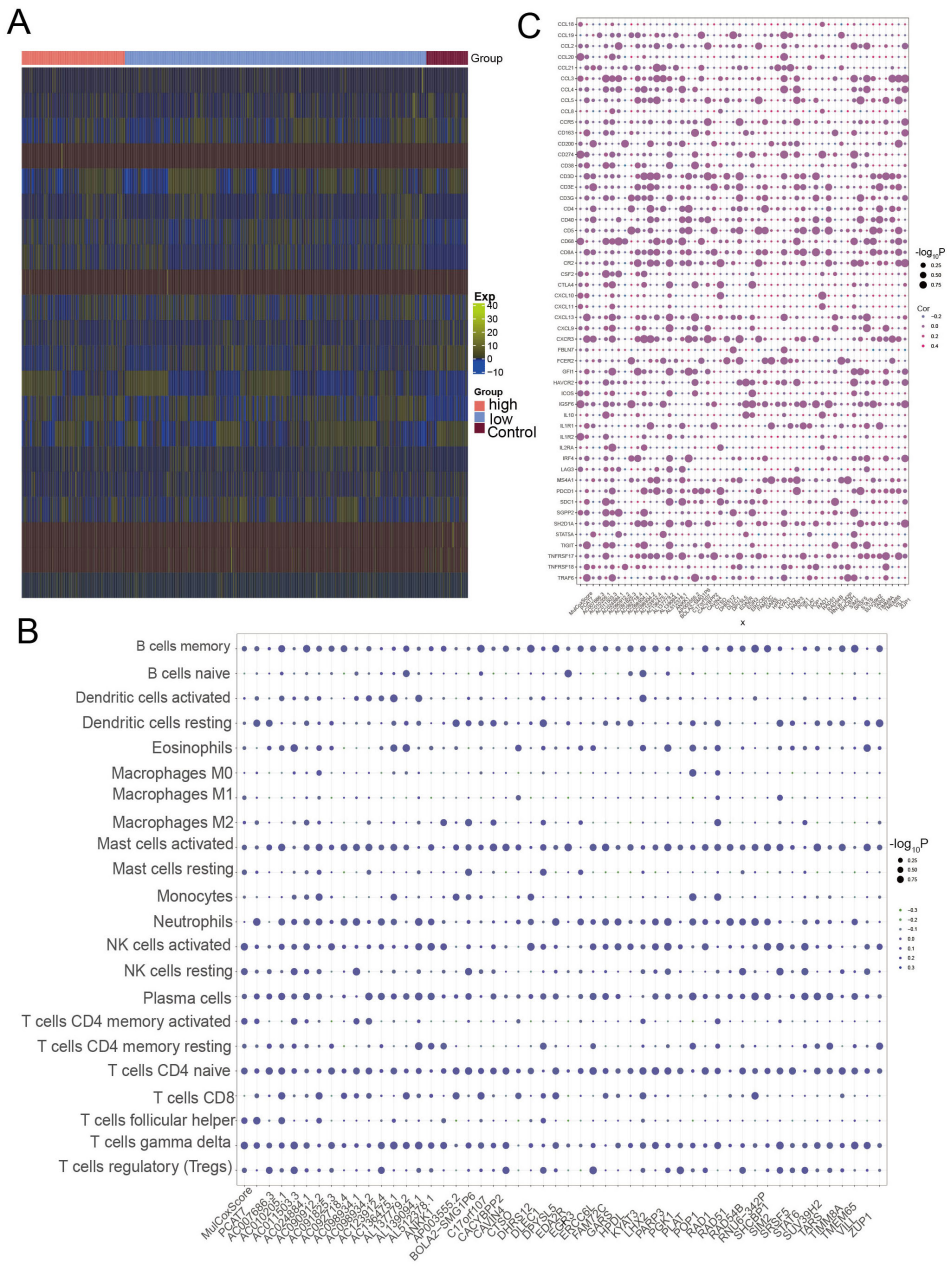
Loss of expression of the PCAT7 gene-based model gene reprogramming in the breast cancer immune microenvironment. **(A)** Heatmap showing the abundance of genes associated with high-low infiltration of immune cells in the control and PCAT7 groups. **(B)** Bubble chart showing the PCAT7 gene-based model and the correlation between the scores and immune cell infiltration abundance. **(C)** Bubble chart showing the significant correlation between the PCAT7 gene-based model and model genes and between several immune checkpoint-related genes and tertiary lymphoid structure marker genes.

A strong correlation was observed between the degree of immune cell infiltration and the progression and prognosis of tumors. The heatmap obtained through immune infiltration analysis showed the infiltration abundance of immune cells in the control group and the high and low-expression groups of the PCAT7 gene (**Figure 6A**). The expression of PCAT7 was positively correlated with the abundance of infiltrating resting mast cells, indicating that PCAT7 promoted the infiltration of the corresponding immune cells. In addition, correlation analysis revealed significant correlations between PCT7-based models and model genes related to immune cells, immune checkpoints, and genes related to tertiary lymphoid structures (**Figures 6B, C**), with RAD51 and PGK1 being particularly significant. Therefore, the PCAT7 gene has a relatively high clinical score and may affect immune cell infiltration and influence the immune microenvironment.

### Upstream regulators of the PCAT7-based clinical model gene set

**Figure 7:**
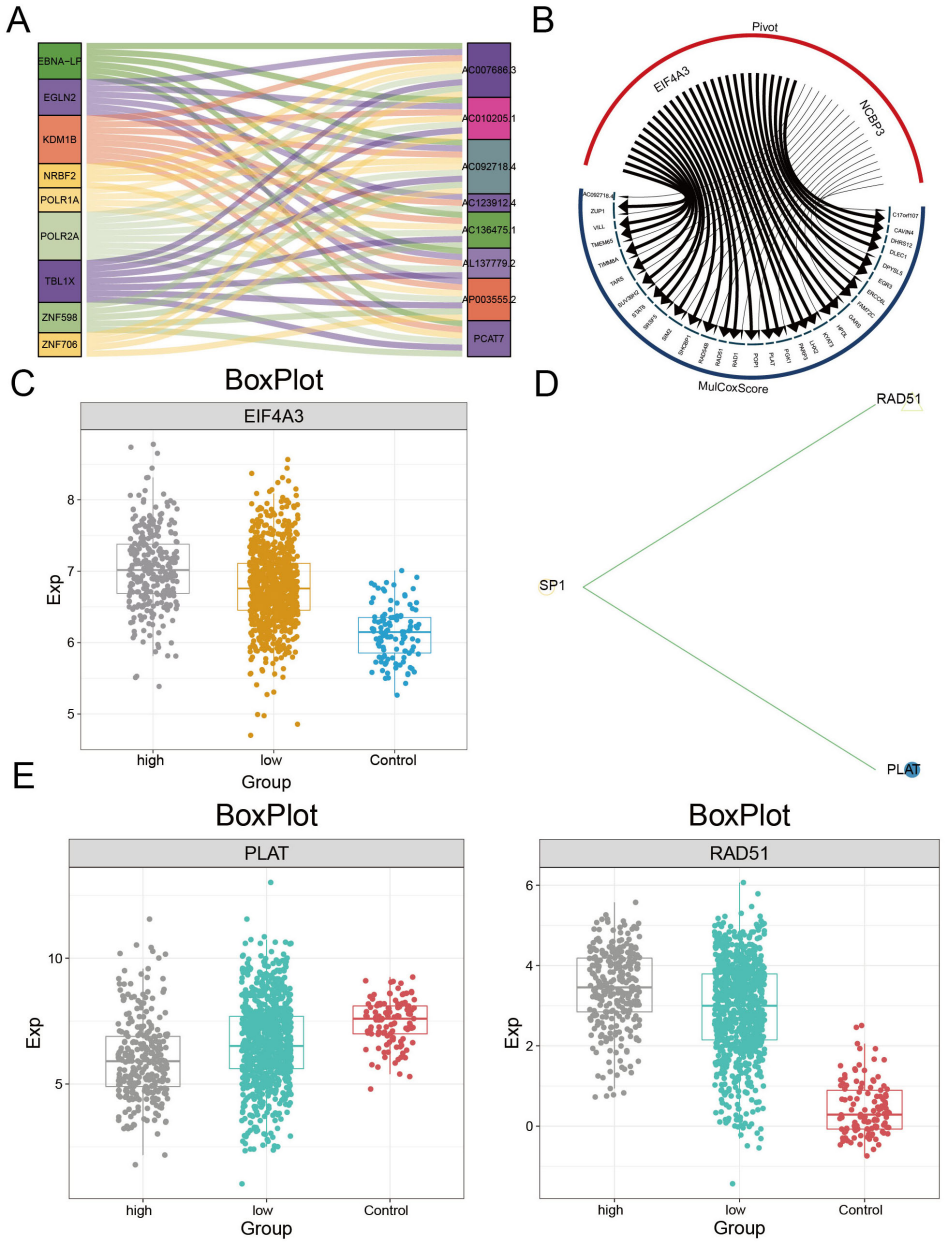
Upstream regulators of the PCAT7 gene-based model gene set. **(A)** Sangi diagram showing the regulatory effect of the lncRNA on the PCAT7 gene-based model gene set. **(B)** The circular network diagram shows the regulatory effect of RBPs on the gene sets of PCAT7-based clinical models. **(C)** Box plot showing the transcription levels of RBPs. **(D)** The circular network diagram shows the regulatory effect of TF on the PCAT7 gene-based clinical model gene set. **(E)** Box plot showing the transcription level of TF.

The upstream regulatory factors of the PCAT7-based model genes were obtained through pivot analysis. This study demonstrated the regulatory effect of an lncRNA on the PCAT7-based model gene set using a Sankey plot (**Figure 7A**), which included lncRNAs such as EGLN2, KDM1B, and TBL1X. The regulatory effect of RBPs on the PCAT7-based clinical model gene set was also demonstrated through a circular network diagram (**Figure 7B**), which included RBPs such as EIF4A3, Its expression level was significantly higher in PCAT7 high expression group and low expression group than that in control group (**Figure 7C**). One TF was SP1, which regulates RAD51 and PLAT in the score gene set (**Figure 7D**), where the RBPs PLAT and RAD51 were significantly differentially expressed in the control, high PCAT7 expression, and low PCAT7 expression BRCA samples (**Figure 7E**).

### Multiomics landscape based on PCAT7 clinical scoring genes

**Figure 8:**
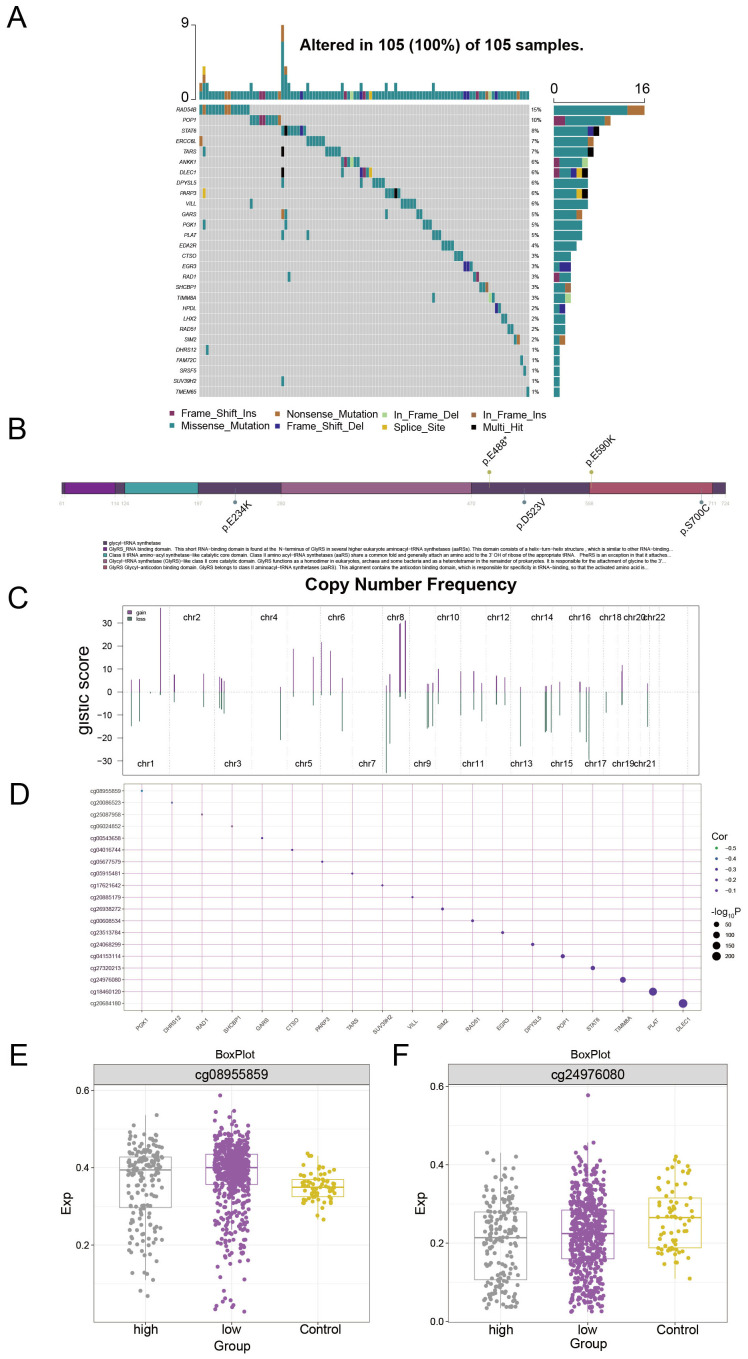
Multiomics landscape based on PCAT7 clinical scoring genes. **(A)** Waterfall diagram showing the mutation landscape (SNP) of PCAT7-based clinical scoring genes in breast cancer. **(B)** Lollipop diagram showing the details of the gene mutations associated with the clinical score based on PCAT7 in breast cancer patients. **(C)** The bar graph shows the copy number spectrum of clinical scoring genes based on PCAT7 in breast cancer. **(D)** The bubble chart shows the correlation between methylation levels and transcription levels of clinical scoring genes based on PCAT7. **(E, F)** Box plot showing the methylation site expression levels in the high-expression group, low-expression group, and control group of clinically expressed genes based on PCAT7.

To further explore the somatic mutation status of PCAT7-based clinical scoring genes and to construct a multiembayment map of the global regulatory network. The results of the waterfall plot showed (**Figure 8A**) that RAD54B had the highest mutation frequency (15%), followed by POP1 (10%), and STAT6 (8%). **Figure 8B** shows the distribution of mutation sites in PCAT7, indicating copy number gains and deletions in the overall regulatory network of PCAT7-based clinical index genes (**Figure 8C**). The correlation between methylation levels and transcription levels of PCAT7-based clinical scoring genes was shown by bubble plots, and we also observed that DLEC1 was associated with PCAT7-based scoring, which was significantly and positively correlated with methylation sites (**Figure 8D**). There was a significant positive correlation with the methylation site (**Figure 8D**). The expression levels of methylation sites within the PCAT7-based clinical scoring genes in the high-expression group, low-expression group and control group are illustrated by box plots, and the methylation sites were highly expressed in the high-expression group, the low-expression group and the control group, respectively, based on the PCAT7-based clinical scoring genes (**Figures 8E, F**).

## Discussion

As one of the most common malignant tumors in the world, BRCA is the most common tumor among women (20). Therefore, further research on BRCA-related genes will help us find additional potential biomarkers and drug targets. The objective of this study was to identify PCAT7-associated module genes in BRCA and use them to establish a clinical indicator model of PCAT7, further exploring the potential role of this model. Data from TCGA database showed that PCAT7 expression levels, as determined by ROC curve and OS curve analysis, were significantly associated with reduced overall survival in breast cancer patients. IHC staining confirmed that the expression of PCAT7 protein was increased in four different subtypes of breast cancer tissues compared with adjacent normal breast tissues. In addition, tumor stage and PCAT7 score were found to be independent risk factors for BRCA. Together, these findings suggest that PCAT7 can serve as a valuable prognostic biomarker for BRCA patients.

DNA replication has a very high impact on cell proliferation, and this study showed that PCAT7 was enriched in biological processes and pathways, especially in DNA replication and the cell cycle, and that there was a significant correlation between DNA replication and BRCA (19). In addition, we also found that PCAT7 was significantly enriched in signaling pathways such as the p53 signaling pathway, cell cycle, and Wnt signaling pathway, which have been proven to be related to cell proliferation, autophagy and apoptosis; tumor cell growth, differentiation and migration; cancer invasion, metastasis, and patient survival (21–23). Multiple studies have shown that genes can affect cancer cell proliferation, apoptosis, and invasion by mediating these signaling pathways (24). In this study, we found that the results were enriched in the p53 signaling pathway and relevant reports suggest that dysregulation of the p53 signaling pathway contributes to the development and progression of cancer (25, 26). Therefore, PCAT7 may affect the proliferation, migration, and invasion of BRCA cells through the p53 signaling pathway, cell cycle, and Wnt signaling pathway. Constructing clinical models offers significant advantages by enabling risk stratification and individualized prognosis prediction for patients. By integrating molecular features with clinical parameters, such models provide quantitative support for treatment decision-making and enhance the implementation of precision medicine. They facilitate early identification of high-risk patients, dynamic assessment of recurrence risk, and personalized intervention planning, thereby offering clear clinical guidance and contributing to more efficient resource allocation and improved treatment outcomes.

According to our research results, high expression of PCAT7 was positively correlated with mast cell infiltration. Research has shown that mast cells originate from CD34+ bone marrow myeloid precursor cells, circulate in the blood, migrate to peripheral tissues, develop and differentiate into mature mast cells under the pressure of tissue-specific chemokines and cytokines, extracellular matrix proteins, and adhesion molecules. Mast cells can promote tumor growth and are considered key mediators of antitumor immunity and modulators of the cancer stroma; they are also related to the intrinsic characteristics of cancer cells (27). Moreover, some research has shown that mast cells play a certain role in BRCA, and they are also enriched in tumor beds and invasion edges of advanced BRCA, especially the luminal subtype (28). Thus, it is hypothesized that elevated levels of PCAT7 result in the infiltration of immune cells, consequently promoting the development of the tumor microenvironment.

In addition, we further analyzed the upstream regulatory factors, including the lncRNAs, RBPs, and TFs, based on the PCAT7 scoring model gene set. Other studies have confirmed that some identified RBPs, such as EIF4A3, play a role in cell proliferation, migration, and tumor invasion (29). Some TFs, such as PLAT, also play important roles in BRCA. Other studies have confirmed that the PLAT may be significantly correlated with the immune status of patients with BRCA by regulating the expression of many immune molecules and influencing immune infiltration in the tumor microenvironment (30). In addition, the results of exploring the multiomics landscape based on PCAT7 clinical score indicate that PCAT7 has a potentially important role in cell communication, which is highly important for further research on BRCA.

In conclusion, our study revealed a close association between PCAT7 high expression and dysregulation of several key genes, and based on this finding, we constructed a clinical prognostic model for BRCA. This innovative model is not only expected to be applied in clinical practice to predict the prognosis of BRCA patients, but also provides a new scientific basis for clinical treatment decisions. Compared with previous studies that focused only on the direct role of BRCA, our study delved into the regulatory role of PCAT7 in BRCA and its key function in gene co-expression network, providing a new perspective to reveal how PCAT7 regulates BRCA.

Despite the remarkable results of our study, there are some limitations. Currently, data assessing the prognostic value of PCAT7 rely mainly on a public database. Future studies are needed to further validate our findings with multicenter, large-scale clinical samples. In addition, additional *in vitro* and *in vivo* functional experiments are needed to confirm the accuracy and reliability of PCAT7 as a prognostic biomarker for BRCA. Further studies on the role of PCAT7 in immune cell infiltration and tumor immune escape in the tumor microenvironment are needed to provide a new perspective and potential therapeutic targets in the field of immunotherapy. Although the constructed model demonstrated good predictive performance in the internal cohort, its generalizability and robustness have not been fully validated due to the lack of independent external validation. Therefore, future studies should incorporate multi-center and more heterogeneous external datasets to further assess the modelsr applicability and reliability across different populations and clinical settings.

## Data Availability

The original contributions presented in the study are included in the article/**Supplementary Material**. Further inquiries can be directed to the corresponding authors.

## Supplementary material

Supplementary Figure 1 Survival curves showing the prognostic ability of recurrent-free survival (RFS) and overall survival (OS) for the module genes most strongly associated with PCAT7 in patients with BRCA.
