# Supplementary figures and images for: Exploration of the clinical prognostic model of BRCA based on PCAT7
Source: Front Oncol. 2025 Jul 30;15:1580858. doi: 10.3389/fonc.2025.1580858 (PMC12343279; doi:10.3389/fonc.2025.1580858)

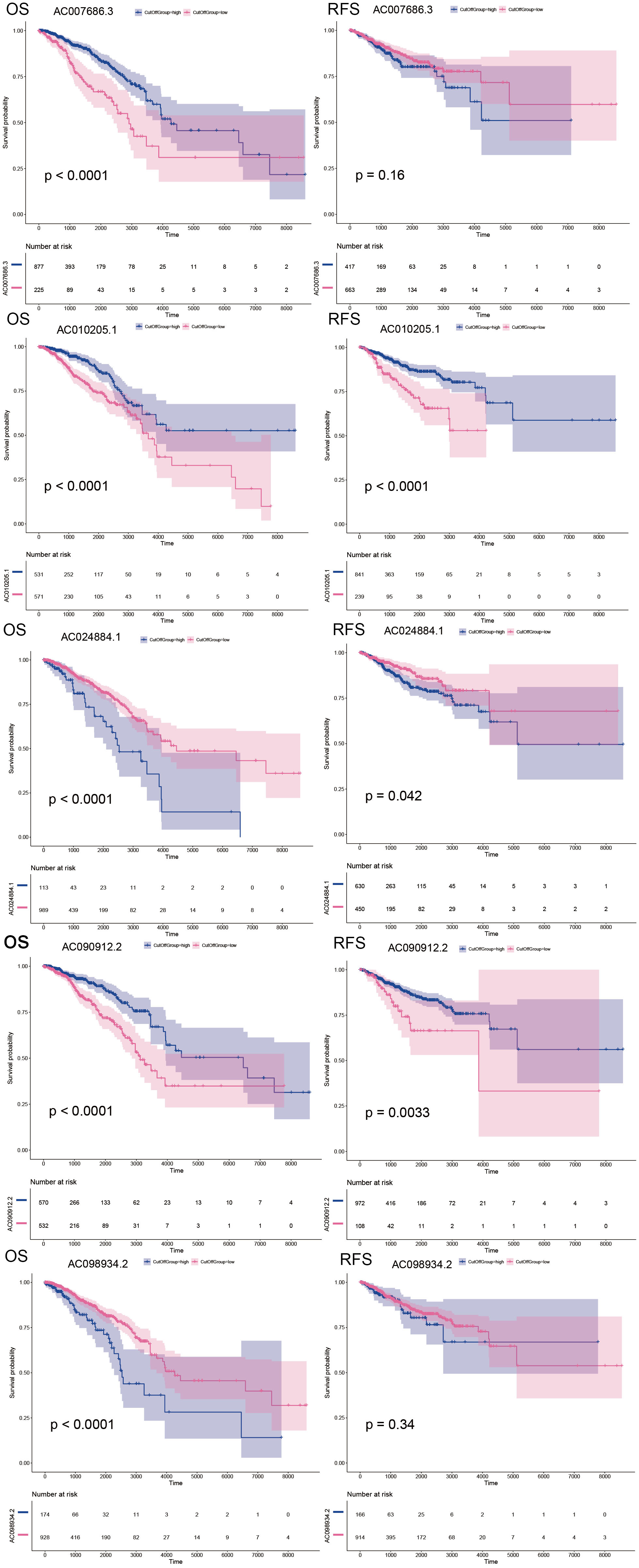

Supplement: Supplementary Figure 1 — Survival curves showing the prognostic ability of recurrent-free survival (RFS) and overall survival (OS) for the module genes most strongly associated with PCAT7 in patients with BRCA. [file Image1.jpeg]
